# Ancient DNA reveals selection acting on genes associated with hypoxia response in pre-Columbian Peruvian Highlanders in the last 8500 years

**DOI:** 10.1038/srep23485

**Published:** 2016-03-21

**Authors:** Lars Fehren-Schmitz, Lea Georges

**Affiliations:** 1 UCSC Human Paleogenomics Lab, Department of Anthropology, University of California, Santa Cruz, Santa Cruz, Ca 95064, USA; 2 Historical Anthropology and Human Ecology, University Goettingen, Goettingen, D-37073, Germany

## Abstract

Archaeological evidence shows that humans began living in the high altitude Andes approximately 12,000 years ago. Andean highlanders are known to have developed the most complex societies of pre-Columbian South America despite challenges to their health and reproductive success resulting from chronic exposure to hypoxia. While the physiological adaptations to this environmental stressor are well studied in contemporary Andean highlanders, the molecular evolutionary processes associated with such adaptations remain unclear. We aim to better understand how humans managed to demographically establish in this harsh environment by addressing a central question: did exposure to hypoxia drive adaptation via natural selection within Andean populations or did an existing phenotype –characterized by reduced susceptibility to hypoxic stress–enable human settlement of the Andes? We genotyped three variable loci within the *NOS3* and *EGLN1* genes previously associated with adaptation to high altitude in 150 ancient human DNA samples from Peruvian high altitude and coastal low altitude sites in a time frame between ~8500–560 BP. We compare the data of 109 successful samples to forward simulations of genetic drift with natural selection and find that selection, rather than drift, explains the gradual frequency changes observed in the highland populations for two of the three SNPs.

Humans residing in the high altitude Central Andes (above 2,500 m.a.s.l.) are directly and indirectly affected by two primary environmental extremes: hypobaric hypoxia and cold. Environments characterized by hypoxic - low concentration of atmospheric oxygen- and cold conditions negatively impact the abundance of resources available to sustain human populations^1^. More directly both extremes act as stressors on the human body affecting biological processes, e.g. reproductive growth, basal metabolic rates, morbidity, and mortality^2^. Despite these obstacles archaeological evidence shows that humans began to live seasonally in the high altitude Central Andes almost immediately after the first settlers arrived on the Pacific littoral of central South America, and permanent residence is evident by ca. 12,000 to 10,000 years ago^1^,^3^. These early inhabitants laid the foundations for what would eventually become the most complex societies of late pre-Columbian South America, namely Wari, Tiwanaku, and Inca, demonstrating the successful adaptation to the harsh environmental conditions of the Andes. However, the molecular evolutionary processes associated with such adaptations remain unclear. It is unknown if the Andes were initially peopled by individuals genetically predisposed with a reduced susceptibility to altitude stress–assuming that individuals suffering from symptoms of e.g. acute hypoxia would have returned to lower altitudes–or whether the descendants of these early pioneers adapted to the environmental stressor over time^1^,^4^.

While prehistoric Andean societies developed behavioral and cultural adaptations to buffer against low biotic productivity and cold^1^, hypoxia constitutes a stress factor acting on human biology that cannot be compensated for by those adaptive mechanisms. Generations of researchers have found that modern Andean populations like the Quechua and Aymara have physiological traits not only distinctive from South American lowland populations but also other highland populations, e.g. in Tibet and Ethiopia^2^. Andean highlanders exhibit elevated hemoglobin concentrations, lower levels of resting ventilation, thoracic expansion, increased lung capacities, a ‘blunted’ hypoxic ventilatory response, higher levels of pulmonary arterial pressure and surprisingly an increased frequency of chronic mountain sickness (CMS) when compared to other highland populations^5^,^6^,^7^,^8^. Thus while most traits ameliorate negative effects of hypoxia, others could represent maladaptation, e.g. CMS. These distinctive phenotypes observed in Andean highlanders and the relatively short time period since the onset of human presence in the high altitude Andes (~480 generations) would suggest strong selective pressures upon putatively beneficial traits^2^,^9^,^10^. With the availability of high-throughput sequencing technology, recent genome-wide scans using single nucleotide polymorphism (SNP) data and whole-genome sequencing data found support for this signature in modern highland populations. Strong signals of selection are reported for a number of candidate genes as *EGLN1, PRKAA1, NOS2*, and *NOS3* associated with the hypoxia-inducible transcription factor (HIF) pathway and nitric oxide (NO) regulation^6^,^11^,^12^,^68^. Also, genes linked to arterial oxygen saturation^13^, increased cardiac vascularization^14^, and transcriptional activity in individuals with CMS^9^ have been identified as being under selection. The results of these studies are compelling, as they identify candidate genes involved in pathways contributing to hypoxia response. But the statistical methods applied in these studies to estimate natural selection are rather indirect as they do nothing more than to detect patterns of novel genomic variation in modern populations that deviate from those expected under neutrality^13^,^14^. This approach can have a reduced sensitivity to detect selection acting on standing variation and has the potential to identify false positives^15^. More importantly, solely relying on modern population data to infer selection in the past leaves the approach prone to bias resulting from past demographic events, e.g. migration and admixture. Considering the effect of demographic change is especially important when studying Native American populations as European contact in the 15–16^th^ centuries inflicted a massive population decline in the Americas, followed by subsequent gene flow from Europe and Africa^16^,^17^. Current paleogenomic methods allow a more direct approach to estimate natural selection as they enable us to monitor allele frequency changes through time under full examination of the respective population history and past episodes of cultural, demographic, and ecological change. Recent ancient DNA studies have set this precedent by not only identifying but also by estimating the intensity of natural selection acting on polymorphisms associated with pigmentation and lactase persistence in European populations^15^,^18^.

Here we test the hypothesis that beneficial traits present in modern Central Andean populations evolved under selection and thus contributed to the increasing survivability and success of societies living in the high altitude environments of the Andes. In doing so we apply a deep-time approach, new for the field of altitude adaptation research. To better understand if and how evolutionary forces might have contributed to the development ofthe genetic diversity of Andean high altitude populations due to the exposure to hypoxia we analyzed three SNPs in two different candidate genes previously associated with response to hypoxic stress (*NOS3*: rs1799983; *EGLN1*: rs1769792, rs1769813) in 150 pre-Columbian individuals dating from ~8,500 to 600 BP. 85 samples derive from archaeological sites in the Peruvian highlands; 65 samples from low altitude coastal sites were included for comparative purposes (Fig. 1; Table 1).

The NOS3 gene on chr7 encodes for endothelial nitric oxide synthase (eNOS), an enzyme catalyzing the synthesis of NO and is i.a. regulated by hypoxia^4^,^19^. NO acts as a vasodilator on pulmonary vessels thereby regulating blood flow and pressure. Typically during acute hypoxic exposure NO levels drop, eventually causing hypoxic vasoconstriction, a condition that is associated with the etiology of high altitude pulmonary edema (HAPE) and also acute mountain sickness (AMS)^4^,^20^. An increased expression of eNOS thus might compensate the hypoxia-induced NO reduction, preventing the described conditions and thereby constitutes both an acclimatization and possible adaption to high altitude stress^4^. The observation that native highland Andean populations exhibit higher levels of NO compared to unadapted lowlanders supports this idea 4,23^69^. NOS3, however, was not identified as a candidate gene for selection in Andean highlanders in a recent study 12. The two NOS3 alleles (rs1799983, G > T at position 894) are common in global populations. This transversion causes a missense mutation (Glu298 to Asp298), thereby reducing gene activity by 50% 24^70^. Several clinical studies have associated the T-allele with an increased susceptibility to hypertension and preeclampsia^21^,^22^. These observations inform our expectation that the G-allele of rs1799983 should increase the fitness of individuals exposed to hypoxic stress.

The two *EGLN1* SNPs (rs1769792 T > G, rs1769813 A > T) provided the highest ranking signals of selection based on locus specific branch length (LSBL) statistics in Andean populations according to a study screening ~900 k SNPs in genes contributing to pathways with known physiological responses to hypoxia, including the HIF pathway, the renin-angiotensin system, and the globin family of genes^23^,^24^. However, it has to be mentioned that a more recent study exploring whole genome data of Andean highlanders did not identify *EGLN1* as being under selection^8^. Ancient and derived alleles of both SNPs are also common in global populations suggesting that the variants precede the peopling of the Americas^25^. The functional role of the *EGLN1* SNPs is less obvious than for *NOS3*. While polymorphisms in this gene have been associated with lowered hemoglobin concentrations in Tibetan populations, a study on Andean women failed to find an comparable association with a hypoxia-related phenotype^6^,^26^. Nevertheless, the positive associations found in other populations, and the significant role of the *EGLN1* gene in the HIF pathway make it a plausible target for our study.

We obtained high confidence genotypes for all three SNPs from 109 ancient Peruvian individuals positively tested for ancient DNA preservation in previous studies. By applying a forward simulation approach modeling drift and selection on standing variation^17^ we directly identify natural selection as causal for allele frequency changes observed for the *NOS3* SNP and one of the *EGLN1* SNPs in the ancient highland populations while the ancient coastal populations exhibit constant allele frequencies over time.

## Results

**Figure 1:**
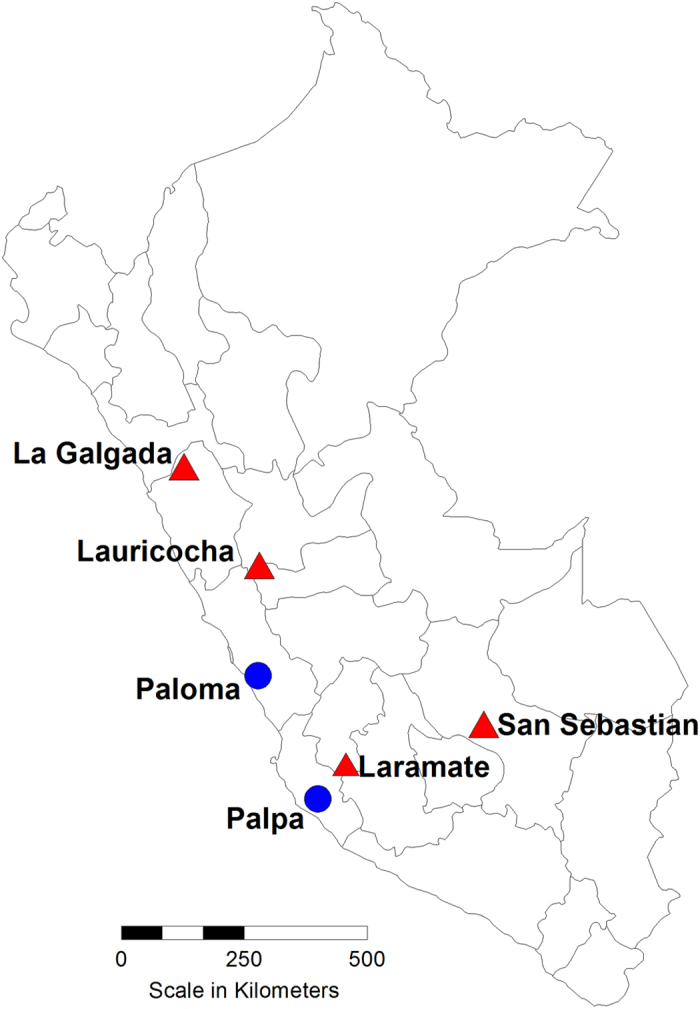
Map of Peru showing the archaeological sites from which the samples derived (red = highland sites; blue = coastal sites). Map was drawn using MapViewer 7.0 (GoldenSoftware, http://www.goldensoftware.com).

The archaeological samples analyzed in this study derived from 14 Peruvian coastal and highland sites (cf. Table 1, Fig. 1). The dates of these sites range from the Early-/Middle Archaic Period (~8500–5500 BP) to late pre-contact sites in the highlands (Late Intermediate Period–Late Horizon, ~1100 AD–1400 AD). The samples analyzed in this study have been part of previous studies investigating mitochondrial and nuclear genetic loci or are currently analyzed in genome wide sequencing projects^27^,^28^,^29^,^30^,^31^,^32^,^33^. All 150 ancient South American samples analyzed here belong to one of the Native American mitochondrial founder haplotypes, determined in previous studies (see Material and Methods, Supplementary Table S1). We used multiplex-PCR combined with a Single Base Extension (SBE) assay to genotype the three altitude adaptation-associated SNPs (rs1799983, rs1769792, rs1769813). Full genotypes for all three SNPs analyzed were successfully obtained for 109 out of the 150 samples (Table 2). Quantitative PCR (qPCR) tests performed prior to the genotyping assay revealed DNA quantity increases with decreasing fragment length by a ratio of at least 1:5 for all samples, as expected for ancient DNA^34^. All negative extraction and PCR controls showed no contamination. In addition to these authentication criteria, downstream analyses were performed only on samples that reproducibly yielded the same genotypes in at least four independent PCR molecules from two independent extracts and where the genotyping results were identical in both labs involved (UCSC-HPG, GoA); these measures exclude/reduce erroneous genotype calls due to allelic dropout.

We compared genotype and allele frequencies for all genotyped SNPs within the ancient populations and with the modern Peruvian reference populations from the 1000Genome Project and the ALFRED database (Table 2). The comparisons revealed that the frequency of the *NOS3* rs1799983 G-allele increases over the ~7,800 year time period for the ancient high altitude population while the EGLN1 rs1769792 G-allele decreases (Table 2). No frequency changes can be observed between the pre-Columbian coastal populations over time but in the case of NOS3, they differ significantly (p < 0.0001) from the modern coastal 1000Genome population from Lima (PEL).

To test if the observed frequency changes over time can be explained by genetic drift we calculated simulation test (ST)^35^, and the Waples test (WT)^36^ statistics implemented in the TAFT software^35^ for the genotype data from coastal and highland temporal transects. This approach based on Bayesian statistics allowed us to take advantage of the sequentially sampled populations rather than only comparing two time periods with each other. To compensate lack of significance due to sample size for the highland populations we merged the Middle with Late Archaic Period populations (MArch_H, LArch_H; n = 14), and the Late Intermediate Period with the Late Horizon populations (LIP_H, LH_H; n = 33) and we excluded the Early Intermediate Period (EIP_H, n = 5) population. Both ST and WT tests confirm that the observed frequency changes for *NOS3* rs1799983 (ST p = 0.0279; WT p = 0.0020) and *EGLN1* rs1769792 (ST p = 0.0401; WT p = 0.0368) over time cannot be explained by drift alone. All tests performed for the coastal populations and *EGLN1* rs1769813 in the highland populations returned no significant results (Table 3). Tests for Linkage Disequilibrium between both *EGLN1* SNPs revealed that they are not in LD for the ancient highland populations (D’ = 0.1811, r^2^ = 0.023, p = 0.0915), while they are found in LD for both the ancient coastal (D’ = 0.3242, r^2^ = 0.0547, p = 0.0234) and the modern 1000 Genome PEL population (D’ = 1.000, r^2^ = 0.9766, p = 0.0000) from Lima which represents a recently admixed population from all over Peru, especially from the highlands. On a global population scale both SNPs are also found to be in LD (D’ = 0.9857, r^2^ = 0.9199, p = 0.0000).

To test if the observed frequency changes in the two hypoxia response-associated alleles of *NOS3* and *EGLN1* in the ancient Andean highlands result from natural selection on standing variation and to quantify the power of potential selection by determining the selection coefficient (s), we performed forward simulations of drift plus selection based on Sverrisdóttir *et al*., allowing uncertainty in ancient allele frequency and population size. We excluded *EGLN1* rs1769813 from these simulations because ST and WT statistics revealed no significant frequency changes over time (Table 3). Because there is no direct phenotype association for the *EGLN1* rs1769792 alleles we tested recessive, dominant and codominant models. For the *NOS3* gene, association studies defined the G-allele either as recessive or codominant depending on the condition tested^4^,^22^,^37^; we thus tested both models. Supplementary Table S2 compiles the p-values calculated for each tested scenario.

**Figure 2:**
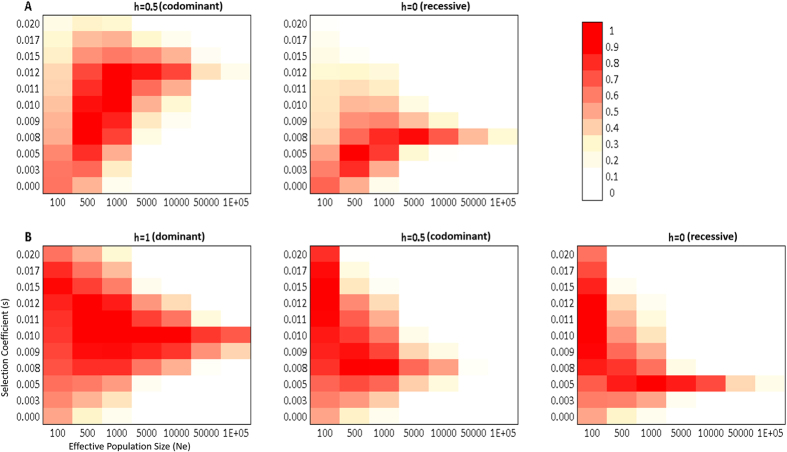
Heatmap illustrating the two-tailed empirical *P* values for the similarity between the observed allele frequencies in the Late Horizon highland population (LH_H) and the distribution of simulated frequencies, given the priors (x = Ne; y = s) modeled between MArch_H and LH_H. (**A**) = NOS3 rs1799983 (codominant, recessive); (**B**) = EGLN1 rs1769792 (dominant, codominant, recessive).

While neutrality (selection coefficient s = 0) for both SNPs was not rejected for this test when assuming very low population sizes (Ne = 100–1000), models considering selection found higher support for all allelic conditions tested (Fig. 2, Supplementary Table S2). The selection coefficients that best explained the observed frequency changes of the *NOS3* rs1799983 G-allele between the Middle- and Late Archaic Period populations (MArch_H, LArch_H; ~8500–4500 BP) and the Late Intermediate Period and Late Horizon (LIP_H, LH_H; ~900–450 BP) or modern Quechua highland population were s = 0.008 (recessive) and s = 0.012 (codominant) respectively (Fig. 2). For the *EGLN1* rs1769792 SNP, the values of selection acting on the G-allele best explaining the observed frequency changes in the highland populations were found to be s = 0.01 (dominant), s = 0.008 (codominant), and s = 0.005 (recessive).

**Figure 3:**
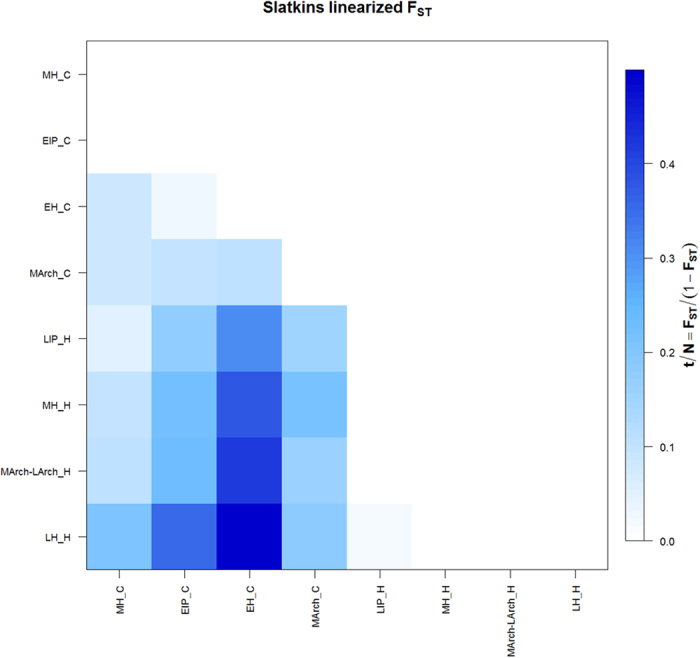
Heatmap visualizing pairwise FST values (Slatkin’s distance) between the ancient populations based on mitochondrial HVRI sequences.

To evaluate if the observed allele frequency changes in the highland populations over time could be explained by population discontinuities we calculated the pairwise genetic distances (FST) between the populations employing the previously published mitochondrial hypervariable region I (HVR) data (Fig. 3). All FST values in between the highland populations were determined as non-significant (p > 0.05) supporting observations of population continuity in the Central Andean highlands during the studied time frame as observed in previous studies^27^,^38^.

## Discussion

Although the physiological responses to hypobaric hypoxia have been well characterized, the underlying genetic basis remains largely unknown. Because of the recent advances in genomics, and a growing number of studies identifying candidate genes involved in particular pathways with hypothesized roles in high-altitude adaptation, it is now possible to directly test the hypothesis that beneficial traits evolved under selection and thus contributed to the survivability and success of societies living in the high altitude environments of the Andes. Here we analyzed putative natural selection candidate loci with standing variation in global populations in ancient Peruvians. We find evidence that selection indeed affected the genetic diversity of Andean highlanders and was ongoing in the high altitude populations during the timespan examined. We did not detect any frequency changes for *EGLN1* rs1769813 previously reported to be under selection in Andean populations by Bigham *et al*.^23^ in the ~8700 year range for which we had samples consistent with the observations of Zhou *et al*.^8^. Interestingly, the allele frequencies observed in the high altitude populations for that SNP resemble those of the ancient coastal populations (cf. Table 2). These observations, combined with a relatively short period of time (~4000 years) between the initial peopling of the Andes and the age of our oldest samples, make it unlikely that selection due to hypoxia-related stress shaped the pattern of variability observed for this locus in Andean highlanders. Other evolutionary forces related to the mode of dispersal of the first settlers arriving in South America should be considered to explain the deviation from the global variability of the *EGLN1* rs1769813 SNP observed in Central Andean populations. Interestingly, both *EGLN1* SNPs tested were only found to not be in LD for the ancient highland populations, while significant non-random associations for the SNPs were found in the ancient coastal, modern Peruvian, and global populations. A possible explanation for this observation could be that a founding effect and subsequent substructuring following the initial divergence into the coastal and the highland populations led to the loss of LD between both markers, and that subsequent selection acting on the *EGLN1* rs1769792 SNP and also more recent admixture between coastal and highland populations after the European contact reestablished LD as seen in the modern Peruvian (PEL) population. To further explore possible scenarios explaining the observation made with regards to changes in LD whole gene data of the ancient specimen for *EGLN1* would be necessary.

The *NOS3* rs1799983 we determined to be under selection has not been identified to be under selection in previous studies of modern DNA^23^ despite the functional association of this polymorphism with physiological traits that can be beneficial in hypoxic environments^4^. By using archaeological human remains to obtain ancient genetic data, we are unable to link the observed genotypes directly to phenotypes. Thus, although we can detect the signal and intensity of selection influencing the *NOS3* G-allele, it is not possible to determine the exact adaptive phenotype/physiological trait to which this genotype contributes. A reduced susceptibility to acute hypoxic stress-related pathologies, namely AMS and HAPE, associated with the G-allele can be correlated with an increased fitness and survivability in high altitude environments^4^. The association of the T-allele with preeclampsia, although not tested in modern Andean populations, and the potential positive effect of increased NO expression on the maintenance of uterine blood flow during pregnancy in hypoxic environments suggest that the *NOS3* G-allele could constitute a reproductive advantage in high altitude populations^22^,^39^.

However, to infer natural selection based on temporal differences in allele frequency it is necessary to assume population continuity^15^. Previous ancient DNA studies, some of which include the present samples, demonstrate clear evidence of population continuity in the high altitude Andes during these archaeological time periods surveyed time frame; these data are based on mitochondrial, autosomal, y-chromosomal markers, and coalescent simulations^28^,^29^,^30^,^32^,^38^,^40^,^41^. Here we calculated pairwise genetic distances (FST) between the studied populations employing the mitochondrial HVR data of the tested samples and found no significant distances between the highland populations supporting the female population continuity observed in the previous studies (Fig. 3; Supplementary Fig. 1). Studies of uniparental inherited and genome-wide genetic markers link these ancient populations to the modern population of the Central Andes^17^,^42^,^43^,^44^. These studies also suggest that geographic structure was established very early in South America, followed by limited gene flow into the Central Andes, until more recent admixture events with other continental populations occurred after European contact^17^. The estimates of the impact of post-Columbian gene flow on the Central Andean gene pool range between 2% to 10% depending on the population^11^,^17^. This study reduces bias resulting from these admixture events by testing pre-contact populations. Our results reveal that the observed genotype and allele frequencies in the late pre-Columbian highland populations largely resemble those of the modern Central Andean populations, supporting high affinities observed between these populations based on other genomic markers^28^,^32^. The significant differences observed for the allele frequencies of the *NOS3* rs1799983 and the *EGLN1* rs1769792 SNPs between the ancient coastal populations and the modern 1000 Genomes population from Lima (PEL) is best explained by a combination of evolutionary factors. An ancient DNA study recently observed population discontinuity along the late pre-contact coast, suggesting larger migrations from the highlands to the coast occurred after ~850 BP, postdating the youngest coastal population tested here^28^. It is important to note that the population of Lima is composed of recent immigrants from rural areas, many of them relocating from the high altitude Andes^45^. The 1000 Genomes Project PEL population thus resembles a general Peruvian and not a specifically coastal population^25^. In addition, the impact of admixture from post-Columbian gene flow must be considered.

The ALFRED database demonstrates a high diversity for *NOS3* rs1799983 in non high altitude South American populations e.g. the in the Amazon, showing both high and low frequencies of the G-allele^46^. While in the first instance this observation appears to contradict the outcome of our study, the processes of early geographic structuring followed by isolation and drift, as mentioned earlier, as well as observed high inter-population differentiation between the Central Andes and other regions of South America, are suitable to explain these frequencies, especially when considering that the effective population size of the initial wave dispersing into South America was very low^17^,^43^,^47^. This suggests that the observed *NOS3* frequency differences in low altitude South America can best be explained by founder effects.

The evolutionary scenarios that found highest support in our study assumed relatively low initial effective population sizes for the highland populations (Ne = 500–5000). This is consistent with the archaeological record showing that the number of initial settlers in the high altitude Andes was considerably low until the stabilization of resource patches and agroeconomic adaptations allowed humans to sustain larger population sizes^1^,^3^. Additionally, constant mobility between the altitude levels of the Andean western slopes persisted in the early phases of settlement^1^. Effects of high altitude stressors on fetal growth and pregnancy would have slowed the demographic development in the Andes until pregnancy-related acclimatization became more widespread^39^,^48^.

The gradual allele frequency change over time observed for of both the *NOS3* rs1799983 and the *EGLN1* rs1769792 SNP allows us to address whether the Andes were initially peopled by individuals genetically predisposed with a reduced susceptibility to hypoxic stress–assuming that individuals suffering from symptoms of AMS, HAPE, CMS would have returned to lower altitudes–or whether the descendants of these early pioneers adapted to the environmental stressor over time^1^,^4^. Our results find support for the latter scenario suggesting a model of niche construction and alteration, where subsequent cultural and behavioral adaptations of the populations living in the Andean highlands constituted a primary buffer acting against environmental stressors, allowing demographic expansions and eventually genetic adaptations to occur^49^. The relevance of this observation for the population history of the high altitude Andes is analogical to that of lactase persistence in European populations, which evolved under comparable evolutionary mechanisms^18^,^49^. However, we have to stress that none of the *EGLN1* SNPs tested in this study have been associated with a hypoxia-related phenotype in Andeans. Additionally, genome wide studies of Andean highlanders did not identify *EGLN1* to be a top ranked candidate for hypoxia induced selection in Andean populations. So, even though we identify a signal of selection in one of the *EGLN1* SNPs tested we have to remark that the involvement of *EGLN1* in the genetic adaptation of Andeans to high altitude stress is not fully supported. The observed selection could result from other environmental factors not considered in this study. An additional factor to be considered in the interpretations of our results is that the demography of the studied populations has not been directly inferred. It is not clear if the observed frequency trajectories have been affected by specific demographic processes (bottlenecks, migrations). To avoid a potential bias resulting from known population bottlenecks resulting from the European contact^16^ we only studied a temporal transect of ancient populations until the end of the pre-Colonial era. Even though we compare our data to modern Central Andean populations, frequencies of these populations are not considered in the simulations. The lack of detection of any significant bottleneck in Native South American populations following the initial peopling until the European contact^16^,^17^ so far suggests that the risk of bias resulting from significant demographic changes during the studied time frame can be seen as minimal.

In the context of other genetic loci identified to be under selection in the more recent history of our species (~10,000 years), the selection coefficients we determined for the *NOS3* rs1799983 and the *EGLN1* rs1769792 SNP (s = 0.008 − 0.012; s = 0.005 − 0.010 depending on the allelic condition considered) are relatively low. For the *LCT* -13,910*T allele, which is strongly associated with lactase persistence in Europeans, recent studies have reported values ranging from s = 0.008 − 0.0795^18^,^50^. The selective advantage of alleles in the *G6PD* gene associated with resistance to malaria in endemic regions has been estimated to range from 0.019–0.049^51^, and the power of selection acting on pigmentation related genes in western Eurasian populations has recently been determined to be between 0.016 and 0.104^15^. Our estimates of selection intensity are within the range (s = 0.002 − 0.029) calculated for other alleles associated with hypoxia response in Tibetan high altitude populations^6^,^52^,^53^. While the genotype-phenotype relationship for the *LCT* and *G6PD* alleles is known to be considerably high (lactase persistence within European populations is almost entirely correlated with the presence of the *LCT* -13,910*T allele), the contribution of alleles associated with hypoxia response to the adapted high altitude phenotype is not completely understood, especially for Andean populations^2^,^6^,^23^. Considering that beneficial adaptations like hemoglobin- or NO-concentration are controlled by complex pathways regulated by a number of genes, the relatively low selection coefficients determined for the two SNPs are not surprising.

In sum, our study reveals direct evidence for natural selection acting on two of three SNPs associated with the adaptation to high altitude stressors and quantifies the strength of selection acting on the specific alleles. We demonstrate the ability of paleogenetics to verify hypotheses solely deriving from modern population studies and the high potential for cooperation between both fields to increase our understanding of our species’ recent evolution. Increased efforts to link genetic diversity to pathways and phenotypes increasing the survivability and reproductive success of these populations are necessary to finally reveal the processes underlying human adaptation to hypoxia in the Andes^6^. This is especially true for the *EGLN1* gene and the two SNPs tested in this study. Even though, we identified selection acting on one of the SNPs, the lack of association for *EGLN1* with a hypoxia-related phenotype in Andean populations also means that other selection inducing factors than hypoxia could be causal for the observed allele frequency changes. At this point the involvement of *EGLN1* in the adaptation of human Andean populations to hypoxia is not fully supported^24^.

Although our study is restricted to three SNPs and the statistical power suffers from low sample size, it offers a simple model to test selective response to environmental stressors. The approach we choose for this study can of course only explore frequency changes in standing variation, and does not allow to detect de-novo mutations that could have contributed to the adaptation of the studied populations. Future ancient DNA studies employing genome-wide sequencing strategies will progressively contribute to the understanding of human adaptation to altitude and have the potential to overcome the discussed limitations of this study.

## Material and Methods

### Ancient samples, DNA extraction, and authenticity

All samples analyzed in this study have been part of previous studies investigating mitochondrial and nuclear genetic loci or are currently analyzed in genome wide sequencing projects^27^,^28^,^29^,^30^,^31^,^32^,^33^. The sites studied where chosen from the collections of the UCSC Human Paleogenomics Lab (UCSC-HPG) so that they represent a diversity of archaeological periods, environmental contexts, and altitude levels. The samples from 150 individuals selected from these sites represent the ones with the best DNA preservation observed in previous investigations (endpoint PCR, qPCR, shotgun sequencing), and therefore were most promising to also contain autosomal DNA. For comparison analysis we collected genotype data from the 1000 Genome Project Lima (PEL) population and the ALFRED database^25^,^46^. All populations analyzed besides their chronological division have been assigned to a basic altitude group (highlands/coast) to compensate low sample size (cf. Table 1).

All pre-PCR analyses were carried out in laboratories entirely dedicated to ancient DNA analysis. All analyses were carried out according to the precautions and contamination prevention strategies described in Fehren-Schmitz *et al*.^28^,^29^. Sample decontamination, preparation and DNA extraction using a silica spin method followed the protocol described in Fehren-Schmitz *et al*.^29^. Mitochondrial haplotypes for all samples were determined previously either by direct sequencing of the HVR1 region and a 26SNP multiplex SBE assay, or hybridization capture of the whole mitochondrial genome followed by high throughput sequencing^29^,^40^,^54^. To further test if the DNA extracted was degraded DNA we tested for asymmetric molecular behavior expected for ancient DNA^34^ performing quantitative PCR, amplifying a 125 bp and a 199 bp nuclear DNA (nDNA) target as described in refs 29,33. Both fragments were amplified using the same upper primer but differing lower primers and have the exact same PCR efficiency. Negative extraction controls and negative PCR controls were employed in this study.

### Analysis of the three hypoxia-response associated SNPs

To determine the genotypes of the *NOS3* and the two *EGLN1* SNPs (rs1799983, rs1769792, 1769813) we developed a multiplex PCR amplifying three targets (95 bp, 86 bp, 92 bp) spanning the sequences containing the SNP of interest coupled with a Single Base Extension (SBE) assay to directly type the SNPs in parallel. Primers for the main PCR and the SBE PCR were designed using the Primer Select software (Lasergene 12.0 package, DNAstar). The minisequencing/SBE primers were designed one base contiguous to the polymorphic site of interest in forward or reverse orientation. An additional polymeric-A tail was added to the 5′ end in order to ensure an effective separation of product lengths during electrophoresis. Primer sequences can be found in Supplementary Table S3. PCR amplification was carried out in a final volume of 12.5 ul containing 6.25 ul Qiagen Multiplex PCR Master Mix (Qiagen, Hilden, Germany), 0.2 uM of each primer, 2–5 ul of DNA template, and DNase free RT-PCR grade H2O (Ambion1, Austin). PCR amplification took place in a C1000 Touch Thermal Cycler (Biorad, Hercules, CA) under the following conditions: initialization at 95 °C for 5 min; 40 cycles at 94 °C for 1 min, 65 °C for 1 min, and 72 °C for 1 min; final elongation at 60 °C for 30 min. PCR success and PCR product quantity were evaluated by gel electrophoresis on 2.5% agarose gels.

To remove excess primers and dNTPs from the previous reaction, the 7.5 ul PCR product of the PCR were first incubated together with 2.5 units (U) rAPid Alkaline Phosphatase (1 U/ul, Roche, Mannheim, Germany), and 1 U Exo I (20 U/ul, New England Biolabs) in a thermocycler for one hour at 37 °C, and following 15 minutes at 75 °C for heat inactivation. Afterwards 1 ul of the purified PCR product was used for the SBE reaction in a final reaction volume of 5 ul consisting of the template, 2.5 ul SNaPshot Ready Reaction Mix (Applied Biosystems, Carlsbad, CA, USA), 0.1 uM SB primer and 20 uM (NH4)2SO4 (Merck, Mannheim, Germany). The ammonium sulfate was added for the suppression of nonspecific peaks in the primer extension reaction^55^. Subsequently, the reaction mixture was treated with 2.5 units of rAPid Alkaline Phosphatase (Roche) at 37 °C for 1 h, and 75 °C for 15 min. to remove the unincorporated fluorescent ddNTPs remaining in the mixture. The Primer extension reaction products were separated and detected by capillary electrophoresis using POP4 polymer on an ABI PRISM 310 Genetic Analyzer (Applied Biosystems). The data was analyzed using GeneScan Analysis Software Version 3.1.2 (Applied Biosystems), and genotypes were determined by confirming the base substitution at the SNP site. Supporting Information Table S1 shows the base substitutions for the three SNP for each sample successfully analyzed.

To ensure authenticity of the amplification results and to prevent false genotype calls due to allelic dropout we performed a minimum of four independent amplifications per DNA extract for each genetic marker analyzed. For each individual we generated at least two independent DNA extracts resulting in a minimum of eight amplification results per marker used to call a consensus genotype. Alleles were only called if they were typed in over 50% of the amplifications. Additionally, 70 samples were tested in a second ancient DNA lab at the University of Goettingen (Goa) employing the same protocols as described for the UCSC-HPG. Samples were discarded if results were inconsistent or there was a risk of false allele determination due to allelic dropout,

### Data Analysis

Allele and genotype frequencies for the *NOS3* and the two *EGLN1* SNPs were obtained using GenAlEx 6.5^56^. Allele frequency changes over time were tested using the simulation test (ST)^35^, and the Waples test (WT)^36^ using the TAFT software^35^. The ST test based on the Bayesian theorem in which the distribution of the distances among sampling frequencies is simulated. The test employs binominal sampling for generation changes and hypergeometric sampling for effective populations and samples as described in Sandoval-Castellanos. WT is a Chi-Square test adjusted to consider gene drift. Both approaches were employed to test the null hypothesis that changes in observed allele frequencies in the sequentially sampled populations are the result of genetic drift and sampling error. The following parameters were used for the above tests: full Bayesian algorithm, Plan I sampling strategy and the respective numbers of generations separating the populations (cf. Table 2). We modeled exponential population growth to a final Ne of 400,000 reflecting ~1/10 of the estimated census population size in the Andes at the point of European Contact^57^. Synchronous allele distributions between coastal and highland populations were additionally compared using Chi-square tests. All tests were two-tailed, and significance level was set at 0.05. The analyses were performed with the SPSS software, version 16 (SPSS, USA). To test for Linkage Disequilibrium (LD) between the two *EGLN1* SNPs we calculated D’ and r^2^ statistics^58^,^59^ for the ancient highland, ancient coastal, and the modern 1000Genome Lima (PEL) population employing the software package Arlequin 3.5^60^. To further explore LD for the two SNPs in global populations we used the online tool LDlink which enables to test LD for SNPs represented in the Phase 3 of the 1000 Genome Project data^61^.

To test if allele frequency changes in the *NOS3* rs1799983 G and the *EGLN1* rs1769792 G-allele and observed in the high altitude time transect can be explained by natural selection and to determine the selection coefficient (s) we used a forward simulation approach based on Wilde *et al*.^15^. Following the descriptions in Wilde *et al*.^15^ and Sverrisdóttir *et al*.^18^ in each forward simulation we first drew the ancestral allele frequency estimate for the oldest population (MArch_H), from which our simulation started from a random Beta (*n*_p_ + 1, *n*_q_ + 1) distribution, where *n*_p_ and *n*_q_ were equal to the number of the respective ancestral and derived allele to reflect uncertainty in the ancient allele frequencies. For both SNPs we then obtained 10,000 MCMC samples by binomial sampling across generations for each combination of priors and allelic condition tested. We used a forward simulation for drift with selection using equation 3.5 from Maynard Smith^62^. We determined the number of generations to forward simulate by defining the number of generations between the oldest population (MArch_H) and the youngest population (LH_H) tested (318 generations, assuming 25 years generation time). The generational distance to the oldest population for the succeeding populations sampled was set by determining the median age of the populations based on the available contextual and radiocarbon data (cf. Table 1). We tested 77 different combinations of priors for each SNP for each allelic condition considered. The variable priors tested were effective population size (Ne) at the time of the ancient sample (Ne = 100 − 100,000) and the selection coefficient (s = 0.00 − 0.02). The upper limits of both priors tested were defined by the empirical observations made during the simulations. We modeled exponential population growth employing the same priors described for the ST and WT test. Subsequently, the simulated distribution of allele frequencies at generation 0 were compared with those observed using the equation 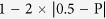, where P is the proportion of simulated modern allele frequencies that are greater than that observed, yielding a two-tailed empirical P value for the observed allele frequency changes for all prior combinations tested^15^,^63^. Simulations were performed in Microsoft Excel 2013.

To explore the population genetic relationship between the tested ancient populations using a neutral genetic marker we employed the 388-bp mitochondrial HVR I sequences (np16,021–np16,408, excluding mutational hotspots np16182 and np16183) reported in previous studies^28^,^31^,^32^,^40^,^64^ from the samples with successfully tested autosomal SNP data. *F*ST values were estimated using the Kimura two-parameter model^65^ using a gamma distribution with shape parameter of 0.205^66^. Both *F*ST and derived Slatkin’s distance^67^ were calculated and visualized using Arlequin 3.5^60^. To compensate lack of significance due to sample size for the highland populations we merged the Middle with Late Archaic Period populations (MArch_H, LArch_H; n = 14), and we excluded the Early Intermediate Period (EIP_H, n = 5) population.

## Additional Information

**How to cite this article**: Fehren-Schmitz, L. and Georges, L. Ancient DNA reveals selection acting on genes associated with hypoxia response in pre-Columbian Peruvian Highlanders in the last 8500 years. *Sci. Rep.* **6**, 23485; doi: 10.1038/srep23485 (2016).

## Supplementary Material

Supplementary Figure 1

Supplementary Dataset 1

## Figures and Tables

**Table 1:** Archaeological sites from which samples derived.

Population Group	Site	Archaeological Period	Date^1^	Altitude (m.a.s.l.)	n
MArch_H	Lauricocha	Early/Middle Archaic Period	8700–8500 BP	4050	2
MArch_C	Palpa	Middle Archaic Period	6000–5000 BP	388	7
	La Paloma	Middle Archaic Period	7000–5500 BP	200	5
LArch_H	La Galgada	Late Archaic Period	5000–4000 BP	1800^2^	11
	Lauricocha	Late Archaic Period	5950 BP	4050	1
EH_H	La Galgada	Formative/Initial Period	4800–4100 BP	1800^2^	4
	Lauricocha	Formative/Initial Period	4640 BP	4050	1
EH_C	Palpa	Formative/Early Horizon	2900–2300 BP	271–388	11
EIP_C	Palpa	Early Intermediate Period	1800–1450 BP	271–388	16
MH_H	Laramate	Middle Horizon	1350–1000 BP	3300–3600	18
LIP_H	Laramate	Late Intermediate Period	900–700 BP	3100–3500	20
LH_H	San Sebastian	Late Horizon/Inca	580–540 BP	2700	13

^1^Dates do not represent the whole archaeological period but the time frames the samples derived from

^2^La Galgada is a ceremonial site in an inner Andean canyon. People buried there most likely arrived from the adjacent residential sites which are all above 2500 m.a.s.l.

**Table 2:** Allele and Genotype frequencies determined for the NOS3 and the two EGLN1 SNPs.

		Generations (T_mod_ - n)	n	NOS3 (rs1799983)					EGLN1 (rs1769792)					EGLN1 (rs1769813)				
				Genotype Freq			Allele Freq		Genotype Freq			Allele Freq		Genotype Freq			Allele Freq	
				G/G	G/T	T/T	G	T	G/G	G/T	T/T	G	T	T/T	T/A	A/A	T	A
Ancient Coast	MArch_C	220	12	0.33	0.58	0.08	0.63	0.37	0.00	0.70	0.30	0.35	0.65	0.00	0.80	0.20	0.40	0.60
	EH_C	98	11	0.36	0.55	0.09	0.64	0.36	0.09	0.64	0.27	0.41	0.59	0.09	0.64	0.27	0.41	0.59
	EIP_C	70	16	0.44	0.38	0.19	0.63	0.37	0.15	0.46	0.38	0.38	0.62	0.13	0.56	0.31	0.41	0.59
Ancient	MArch_H	340	2	0.50	0.50	0.00	0.75	0.25	0.50	0.50	0.00	0.75	0.25	0.50	0.50	0.00	0.75	0.25
Highlands	LArch_H	180	12	0.50	0.42	0.08	0.71	0.29	0.60	0.30	0.10	0.75	0.25	0.40	0.20	0.40	0.50	0.50
	EH_H	136	5	0.60	0.40	0.00	0.80	0.20	0.40	0.40	0.20	0.60	0.40	0.40	0.20	0.40	0.50	0.50
	MH_H	47	18	0.78	0.22	0.00	0.89	0.11	0.28	0.56	0.17	0.56	0.44	0.22	0.50	0.28	0.47	0.53
	LIP_H	30	20	0.90	0.05	0.05	0.93	0.07	0.28	0.50	0.22	0.53	0.47	0.20	0.60	0.20	0.50	0.50
	LH_H	22	13	0.92	0.08	0.00	0.96	0.04	0.08	0.85	0.08	0.50	0.50	0.15	0.77	0.08	0.54	0.46
ALFRED_QUE	Mod_H	0	46	n.d.	n.d.	n.d.	0.93	0.07	n.d.	n.d.	n.d.	n.d.	n.d.	n.d.	n.d.	n.d.	n.d.	n.d.
1kGenomes_PEL	Mod_C	0	85	0.82	0.18	0.00	0.92	0.08	0.35	0.36	0.28	0.53	0.47	0.36	0.35	0.28	0.54	0.46

**Table 3:** Simulation Test (ST) and Waples Test (WT) results for the sequential comparisons of all three SNPs in the highland populations and the coastal populations.

	NOS3 (rs1799983)		EGLN1 (rs1769792)		EGLN1 (rs1769813)	
	ST^1^	WT^2^	ST	WT	ST	WT
Coast^3^	0.9919	0.9957	0.8033	0.8676	0.7512	0.5916
Highlands^4^	**0.0279**^5^	**0.0020**	**0.0401**	**0.0368**	0.6164	0.7482

^1^ST = Simulation-Test p-value.

^2^WT = Waples-Test p-value.

^3^Comparison = MArch_C–EH_C–EIP_C.

^4^Comparison = MArch_H & LArch_H–MH_H–LIP_H & LH_H–Quechua_Modern.

^5^Significant P-values are in bold (significance level ≥0.05).
