# Supplementary material for: Ancient DNA reveals selection acting on genes associated with hypoxia response in pre-Columbian Peruvian Highlanders in the last 8500 years
Source: Sci Rep. 2016 Mar 21;6:23485. doi: 10.1038/srep23485 (PMC4800713; doi:10.1038/srep23485)
Supplement: Supplementary Figure 1 [file srep23485-s1.pdf]

Supplementary Materials

Ancient DNA reveals selection acting on genes associated with hypoxia response in pre-Columbian Peruvian Highlanders in the last 8500 years

Authors: Lars Fehren-Schmitz[1*], Lea Georges[1,2],

* Corresponding Author

[1] UCSC Human Paleogenomics Lab, Department of Anthropology, University of California, Santa Cruz, Santa Cruz, Ca 95064, USA

[2] Historical Anthropology and Human Ecology, University Goettingen, Goettingen, D-37073, Germany

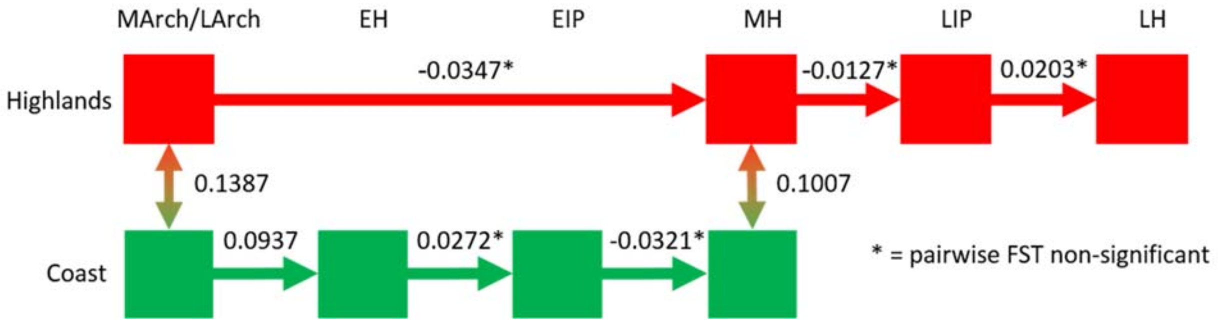

Supplementary Figure 1. Pairwise FST values based on mitochondrial HVR1 sequence data calculated in between the studied ancient Peruvian highland (red) and coastal (green) populations. (MArch/LArch = Middle- and Late Archaic Period, ~8000-5000 BP; EH = Early Horizon, ~4000-2200 BP; EIP = Early Intermediate Period, ~2200-1400 BP; MH = Middle Horizon, ~1400-900 BP; LIP = Late Intermediate Period, 900-600 BP; LH = Late Horizon, 600-500 BP).
